# Highly pathogenic avian influenza H5N1 virus delays apoptotic responses via activation of STAT3

**DOI:** 10.1038/srep28593

**Published:** 2016-06-27

**Authors:** Kenrie P. Y. Hui, Hung Sing Li, Man Chun Cheung, Renee W. Y. Chan, Kit M. Yuen, Chris K. P. Mok, John M. Nicholls, J. S. Malik Peiris, Michael C. W. Chan

**Affiliations:** 1 Centre of Influenza Research and School of Public Health, LKS Faculty of Medicine, The University of Hong Kong, Hong Kong SAR, China; 2 Department of Paediatrics, Faculty of Medicine, The Chinese University of Hong Kong, Hong Kong SAR, China; 3 The HKU-Pasteur Research Pole, School of Public Health, LKS Faculty of Medicine, The University of Hong Kong, Hong Kong SAR, China; 4 Department of Pathology, LKS Faculty of Medicine, The University of Hong Kong, Queen Mary Hospital, Hong Kong SAR, China

## Abstract

Highly pathogenic avian influenza (HPAI) H5N1 virus continues to pose pandemic threat, but there is a lack of understanding of its pathogenesis. We compared the apoptotic responses triggered by HPAI H5N1 and low pathogenic H1N1 viruses using physiologically relevant respiratory epithelial cells. We demonstrated that H5N1 viruses delayed apoptosis in primary human bronchial and alveolar epithelial cells (AECs) compared to H1N1 virus. Both caspase-8 and -9 were activated by H5N1 and H1N1 viruses in AECs, while H5N1 differentially up-regulated TRAIL. H5N1-induced apoptosis was reduced by TRAIL receptor silencing. More importantly, STAT3 knock-down increased apoptosis by H5N1 infection suggesting that H5N1 virus delays apoptosis through activation of STAT3. Taken together, we demonstrate that STAT3 is involved in H5N1-delayed apoptosis compared to H1N1. Since delay in apoptosis prolongs the duration of virus replication and production of pro-inflammatory cytokines and TRAIL from H5N1-infected cells, which contribute to orchestrate cytokine storm and tissue damage, our results suggest that STAT3 may play a previously unsuspected role in H5N1 pathogenesis.

Highly pathogenic avian influenza (HPAI) H5N1 viruses continue to transmit zoonotically with mortality around 53% and pose a pandemic threat^1^,^2^. Early antiviral therapy improves clinical outcome but even commencement of antivirals within 2–4 days of clinical onset does not assure survival^1^,^3^. Acute respiratory distress syndrome is the primary cause of respiratory failure associated with severe influenza disease but there is presently no effective therapy to reverse this pathology^2^,^3^,^4^. The mechanisms underlying the pathogenesis of human H5N1 disease remains uncertain. Viral antigen was detected in alveolar epithelium in autopsy lung of H5N1 infected patient, indicating that the alveolar epithelium is the major site of HPAI H5N1 influenza virus infection in humans^5^. Using *ex vivo* lung culture, we have confirmed that alveolar epithelial cells are a primary target cells for H5N1 virus infection^6^,^7^. Both alveolar type I and II epithelial cells support the replication of HPAI H5N1 virus^8^.

Limited available autopsy studies in H5N1 infected patients have shown evidence of apoptosis in human alveolar epithelial cells^5^. Apoptosis is a common phenomenon observed in influenza infected cells. Tissue damage can result from direct induction of apoptosis by infection or indirect apoptosis caused by inflammatory mediators and death ligands secreted from infected cells. Earlier studies have shown that influenza virus induces apoptosis *in vitro*^9^,^10^,^11^ and *in vivo*^12^. Influenza-induced apoptosis has been reported in various type of cells, such as retinal pigment epithelial cells^13^, bronchial epithelial cells^14^,^15^, nasopharyngeal, lung, and intestinal epithelial cells^16^,^17^, swine airway epithelial cells^18^, natural killer cells^19^ and in *ex vivo* cultures of human lung^20^.

We have previously reported that H5N1 virus triggered apoptosis in primary human peripheral blood monocyte-derived macrophages was delayed in comparison with seasonal influenza infected cells. We also demonstrated that the activation of the intrinsic pathway was responsible for apoptosis^21^. Human H5N1 influenza virus induced higher expression of TRAIL and triggers the apoptosis of neighboring cells through the cell-cell interaction^22^. There are reports of the differential expression of apoptosis-related genes induced by H1N1 and H5N1 viruses in human lung epithelial cells and in mice^23^,^24^. However the signaling pathways responsible for such differences between seasonal and H5N1 influenza are poorly explored.

STAT3 has been found to be constitutively activated in various types of cancer cell lines and tumors. Compelling evidence has been reported on the activation of STAT3 in cancer formation and STAT3 has become a target for inducing apoptosis in solid and hematological tumors^25^,^26^,^27^. The activation of STAT3 leads to the dysregulation of a number of biological processes, including cell cycle control, apoptosis genes and genes for invasion, metastasis, and angiogenesis^26^. However, there is limited information on the role of STAT3 on apoptosis induction by influenza virus in human respiratory epithelium.

## Results

### Infection and replication of influenza A virus in human bronchial (HBECs) and alveolar epithelial cells (AECs)

**Figure 1:**
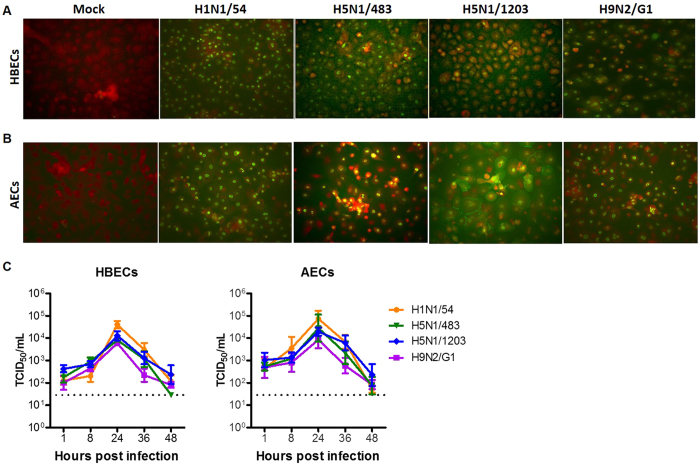
Influenza virus infection and replication in human bronchial and alveolar epithelial cells. (**A,B**) The cells were infected with mock, influenza H1N1/54, H5N1/483, H5N1/1203, or H9N2/G1 for 24 hours, fixed, and stained with antibody against influenza M and NP proteins (green) with cytoplasm stained in red. Representative pictures of HBECs (**A**) and AECs (**B**) are shown at a magnification of 200×. (**C**) Viral replication kinetics in HBECs and AECs were measured by TCID_50_ assay at indicated time points. Results are means ± SEM of at least three biological replicates. Horizontal dotted line denotes the detection limit of the viral titration assay.

HBECs and AECs were infected with influenza A viruses (H1N1/54, H5N1/483, H5N1/1203, H9N2/G1) at a multiplicity of infection of 2 (MOI = 2) so as to ensure most of the cells were infected in synchrony. Under these conditions, around 90% of cells were infected by each of the influenza viruses studied (Fig. 1A,B). Virus replication kinetics of the different influenza A viruses tested (H1N1/54, H5N1/483, H5N1/1203 and H9N2/G1) was similar in both cell types *in vitro* (Fig. 1C) with productive replication indicated by the increase in viral titers during the first 24 hpi. These results indicate that both HBECs and AECs were susceptible to influenza A infection *in vitro*.

### H5N1 delayed the onset of apoptosis in human HBECs and AECs

**Figure 2:**
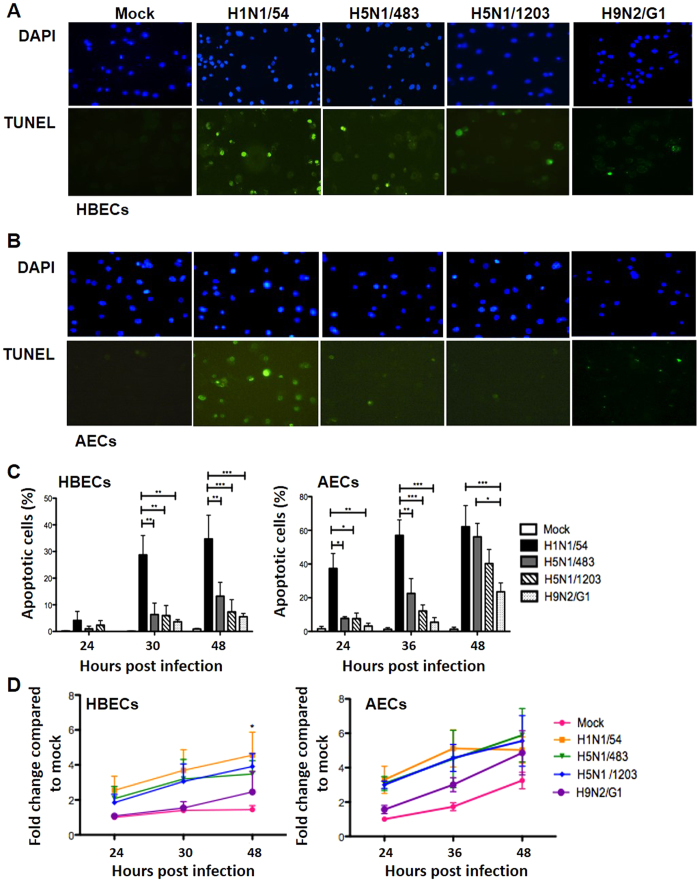
Apoptosis and necrosis in HBECs and AECs after influenza infection. Representative pictures of apoptotic cells (in green) and nucleus (in blue) are shown in (**A**) HBECs at 30 hpi and (**B**) AECs at 24 hpi with magnification of 400×. (**C**) Percentage of apoptotic cells at indicated time points upon mock and influenza virus infection in HBECs and AECs are shown. Percentages of apoptosis was derived from the number of TUNEL staining positive cells over the number of DAPI stained nuclei. Results are means ± SEM of at least three biological replicates, *p < 0.05, **p < 0.01, ***p < 0.001 calculated by two way ANOVA with Bonferroni’s post-test. (**D**) LDH level in the culture supernatants of cells with mock or influenza infection were measured in HBECs and AECs. Results are means ± SEM of at least three biological replicates, *p < *0.05* compared to corresponding mock and calculated by two way ANOVA with Bonferroni’s post-test.

The apoptotic response induced by influenza viruses in human respiratory epithelial cells was assessed using TUNEL staining (Fig. 2A,B). We quantified the apoptotic response by calculating the percentage of TUNEL positive cells versus total number of cell with DAPI staining (Fig. 2C).

There was no significant differences in the percentage of apoptotic cells between the virus and mock infected HBECs at 24 hpi (Fig. 2C). Differential induction of apoptosis was observed at 30 and 48 hpi (Fig. 2C) with H1N1/54 virus inducing the highest percentage of cells undergoing apoptosis among the four viruses in HBECs (Fig. 2C).

In human AECs, apoptosis was observed as early as 24 hpi in H1N1/54 virus infected cells but was significantly lower with the other three viruses tested (Fig. 2C). The percentage of apoptotic cells in H5N1 and H9N2 virus infected cells increased by 48 hpi but H9N2 virus infected cells still had significantly lower induction of apoptosis compared with other viruses. We observed a similar delayed pattern of apoptosis induction by influenza H5N1 viruses (H5N1/483 and H5N1/1203) and H9N2 virus (H9N2/G1) in both cell types. However, the kinetic of apoptosis induction by influenza viruses was slower in the HBECs when compared to AECs. The measurement of apoptosis was repeated by immunohistochemical staining of cleaved-caspase 3 at 48 and 72 hpi. We observed similar trend of apoptosis in both HBECs and AECs at 48 hpi (data not shown). The percentages of apoptotic cells induced by H1N1/54, H5N1/483 and H5N1/1203 dropped at 72 hpi compared to 48 hpi in AECs. H1N1/54-induced apoptosis dropped at 72 hpi while H5N1/483 and H5N1/1203 induced low level of apoptosis at 72 hpi in HBECs (data not shown).

Necrosis induced by these influenza A viruses and mock infected cells was assessed by the level of lactate dehydrogenase (LDH) in the cell culture supernatants of HBECs and AECs (Fig. 2D). In contrast to apoptosis, necrosis in HBECs and AECs infected with H1N1/54, H5N1/483 and H5N1/1203 viruses were comparable and they induced more necrosis than H9N2/G1 infected HBECs, although without statistical significance (Fig. 2D). Comparable induction of necrosis by H1N1/54, H5N1/483 and H5N1/1203 viruses was confirmed with trypan blue exclusion assay at 24 and 48 hpi (data not shown).

### Influenza H1N1 virus induced more activation of caspase 3 in AECs

Since the patterns of delayed apoptosis in AECs and HBECs were similar and AECs are the main target cells of influenza infection^7^,^28^, we used AECs and seasonal H1N1/54 and HPAI H5N1/483 viruses as representatives of early- and late-apoptosis inducing viruses respectively, in the subsequent mechanistic studies. We monitored the activation of caspase 3 by the two viruses in terms of the expression of proteolytic cleaved products at 17 kDa and 19 kDa^29^.

**Figure 3:**
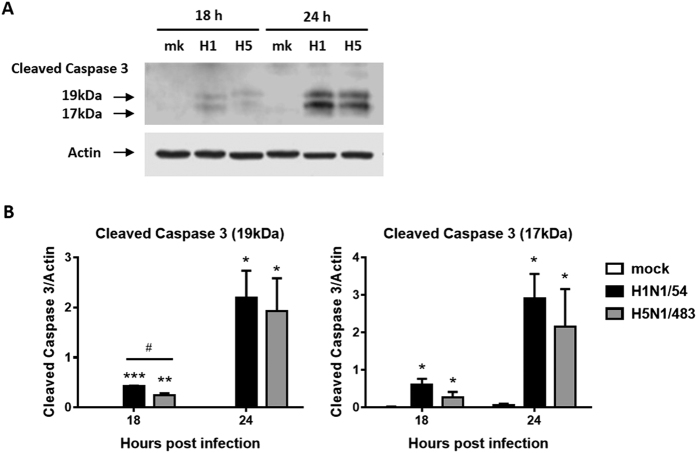
H1N1/54 virus activates more caspase 3 than H5N1/483 virus in AECs. (**A**) Expression of cleaved caspase 3 was detected by Western blotting. Results are from a representative experiment. Actin was used as loading control. (**B**) The relative intensity of bands at 19 kDa and 17 kDa was calculated with corresponding actin and plotted. Data are means ± SEM of three biological replicates. *p < 0.05; **p < 0.01; ***p < 0.001, compared to mock infected samples calculated by two way ANOVA with Bonferroni’s post-test; ^#^*p* < 0.05, compared between H1N1/54 and H5N1/483 calculated by two-tailed Student’s t-test.

H1N1/54 virus induced a significantly higher level of cleaved caspase 3 (19 kDa) than H5N1/483 virus infected cells at 18 hpi but there was no significant difference between the two viruses at 24 hpi (Fig. 3A,B). Similar pattern was observed in the expression of 17 kDa fragment. In general, H1N1/54 virus activated caspase 3 earlier than H5N1/483. There was no activation of caspase 3 in mock infected cells at both 18 and 24 hpi (Fig. 3A,B).

### Influenza H1N1 and H5N1 viruses activate caspase 8 and 9 to induce apoptosis

**Figure 4:**
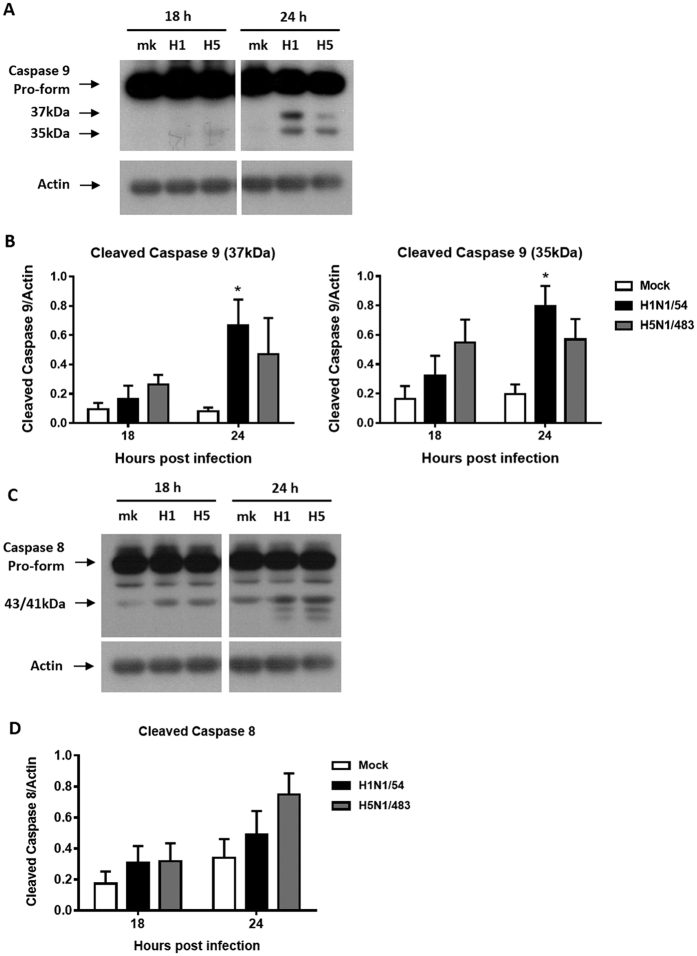
Activation of caspase 9 and 8 by H1N1/54 and H5N1/483 viruses in AECs. Expression of cleaved caspase 9 (**A**) and cleaved caspase 8 (**C**) was detected by Western blotting. Representative cropped blots at 18 and 24 h are shown. All the samples on the blots presented were run in the same gel and under the same experimental conditions. The full-length blots of cleaved caspase 9 and caspase 8 with actin are presented in Supplementary Figs S1 and S2, respectively. The relative intensity of bands at 37 kDa and 35 kDa of cleaved caspase 9 (**B**) and cleaved caspase 8 (**D**) was calculated with corresponding actin and plotted. Data are means ± SEM of three biological replicates. *p < 0.05, compared to mock infected cells at corresponding time point calculated by two way ANOVA with Bonferroni’s post-test.

We further investigated the signaling pathways leading to apoptosis by measuring the activation of caspase 8 and 9. Both H1N1/54 and H5N1/483 viruses activated caspase 9 in AECs (Fig. 4A,B; see Supplementary Fig. S1 for full-length blots online). Levels of the cleaved form of caspase 9 was significantly higher in H1N1/54 infected cells than in H5N1 infected cells at 24 hpi (Fig. 4A,B).

Furthermore, the activation of caspase 8 was similar between the H1N1/54 and H5N1/483 viruses at 18 hpi (Fig. 4C,D; see Supplementary Fig. S2 for full-length blots online). However, there was a trend of higher expression level of cleaved caspase 8 in AECs infected by H5N1/483 (0.824 normalized with actin) than that by H1N1/54 (0.642 normalized with actin) at 24 hpi (Fig. 4C), although there was no statistical significance in pooled results from different donors (Fig. 4D). Taken together the above findings, we conclude that both H1N1/54 and H5N1/483 viruses induced both intrinsic and extrinsic apoptosis pathways.

### H5N1-induced apoptosis is partially dependent on the up-regulation of TRAIL

TRAIL is a death receptor ligand, which is an upstream signal of caspases for the induction and activation of apoptosis. Since TRAIL mRNA was up-regulated in H5N1/483 infected human macrophages in previous report^22^, in this study the expression of TRAIL was evaluated in both influenza H1N1/54 and H5N1/483 infected human AECs.

**Figure 5:**
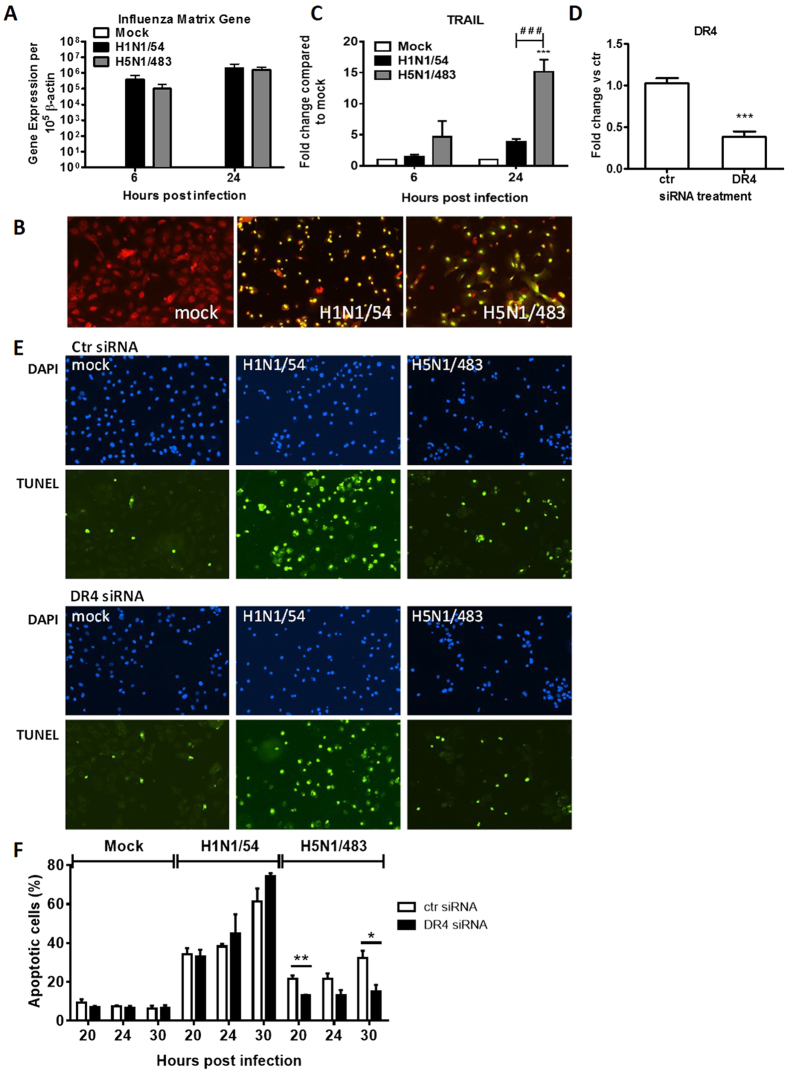
H5N1/483 but not H1N1/54-induced apoptosis is partially dependent on the up-regulation of TRAIL in AECs. (**A**) The mRNA expression of influenza matrix gene and (**B**) immunofluorescent staining of influenza M and NP proteins (green) in AECs at 24 hpi were shown. (**C**) The mRNA expression of TRAIL in AECs was measured at 6 and 24 hpi. Data are means ± SEM of three biological replicates. ***p < *0.001*, compared with corresponding mock calculated by two way ANOVA with Bonferroni’s post-test. ^###^*p* < 0.001, compared between H1N1/54 and H5N1/483 calculated by two-tailed Student’s t-test. (**D–F**) AECs were transfected with control (ctr) or DR4 siRNAs. (**D**) The knock-down efficiencies were assessed by qPCR. Data are means ± SEM of three biological replicates, ***p < *0.001*, compared between ctr and DR4 siRNA transfected cells calculated by two-tailed Student’s t-test. (**E**) 24 h after transfection, the cells were infected with mock, H1N1/54, or H5N1/483 at MOI 2 and subjected to TUNEL assay at 20, 24, and 30 hpi. Micrographs of apoptotic cells (in green) and nucleus (in blue) at 30 hpi are shown. (**F**) Percentages of apoptotic cells induced by the two viruses in siRNA transfected cells were shown. Data are means ± SEM of three biological replicates, *p < 0.05; **p < *0.01*, compared between ctr and DR4 siRNA transfected cells infected with the same virus at indicated time points calculated by two-tailed Student’s t-test.

The mRNA expression of influenza matrix (M) gene was used to evaluate virus replication competence in AECs at 6 and 24 hpi and its mRNA level was comparable between influenza H1N1/54 and H5N1/483 infected human AECs (Fig. 5A). Immunofluorescence staining of influenza M and NP protein showed that almost all AECs were infected at 24 hpi by influenza H1N1/54 or H5N1/483 viruses (Fig. 5B). Fold change of TRAIL mRNA expression compared to mock infected cells was plotted (Fig. 5C). H5N1/483 started to induce the mRNA expression of TRAIL at 6 hpi and this was highly up-regulated at 24 hpi. The mRNA expression of TRAIL was significantly higher in H5N1/483-infected cells compared to H1N1/54-infected cells (Fig. 5C). However, there was no up-regulation of the receptor of TRAIL—death receptor 4 (DR4) by the influenza viruses (data not shown).

In order to monitor the relation between virus-induced TRAIL and apoptosis induction, we transiently knocked-down DR4 by using siRNA. The knock-down efficiency was 62% compared to scramble knock-down using non-targeting siRNA (ctr) (Fig. 5D). siRNA transfected AECs were infected with H1N1/54 or H5N1/483 and the apoptotic responses were measured by TUNEL assay. There was a significant reduction of apoptotic cells (around 39–53% reduction) in DR4-knock-down cultures compared to ctr-knock-down cultures after H5N1/483 infection but not after H1N1/54 infection (Fig. 5E,F). These findings suggest that the induction of apoptosis by H5N1/483 is, at least in part, dependent on the expression of TRAIL, and since H1N1/54 did not induce high level of TRAIL, the DR4 knock-down did not affect the apoptosis induction in H1N1/54 infected cells.

### Activation of STAT3 is involved in the delay of apoptosis by H5N1

**Figure 6:**
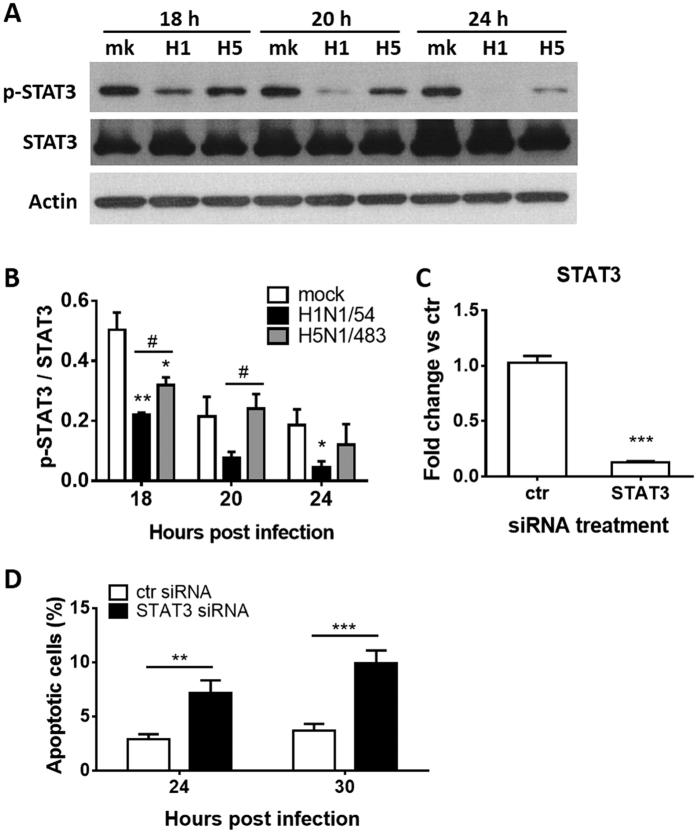
H5N1/483 inhibits apoptosis by activation of STAT3. (**A**) Phosphorylation of STAT3 (p-STAT3) by mock, H1N1/54 and H5N1/483 infection in AECs was measured by Western Blotting. Representative blots of p-STAT3, and STAT3 proteins are shown. Actin is shown as loading control. (**B**) The activation of STAT3 in terms of phosphorylation was normalized with STAT3 protein. Results are means ± SEM of three biological replicates. *p < 0.05; **p < *0.01*, compared with mock infected cells calculated by one way ANOVA and Bonferroni’s post-test. ^#^*p* < *0.05*, compared between H1N1/54 and H5N1/483 infected cells calculated by two-tailed Student’s t-test. (**C,D**) AECs were transfected with control (ctr) or STAT3 siRNAs. (**C**) The knock-down efficiencies were assessed by qPCR. Data are means ± SEM of three biological replicates. ***p < *0.001*, compared between ctr and STAT3 siRNA transfected cells calculated by two-tailed Student’s t-test. (**D**) 24 h after transfection, the cells were infected with H5N1/483 at MOI 2 and subjected to TUNEL assay at 24, and 30 hpi. Apoptosis was assessed by TUNEL staining. The percentages of apoptotic cells induced by H5N1/483 are shown. Data are means ± SEM of three biological replicates, **p < *0.01*; ***p < *0.001*, compared between ctr and STAT3 siRNA transfected cells infected with H5N1/483 at indicated time points calculated by two-tailed Student’s t-test.

Constitutive activation of STAT3 has been observed in various tumor cell lines and STAT3 activation is associated with cell survival and induction of growth signaling pathways. Treatment to inhibit the activation of STAT3 leads to inhibition of cell survival and growth signaling pathways in various tumor cell lines^27^,^30^. Therefore, we further investigated the involvement of STAT3 in apoptosis induction by influenza viruses. Both H1N1/54 and H5N1/483 viruses inhibited the activation of STAT3 compared to mock infected cells (Fig. 6A,B). However, H5N1/483 infection led to more activation of STAT3 than H1N1/54 did and there was a decrease of activated STAT3 with time in H5N1/483 infected cells. By using siRNA silencing, we achieved 87.5% knock-down of STAT3 (Fig. 6C). STAT3 knock-down cells had significantly more apoptosis than control knock-down cells during H5N1/483 infection (Fig. 6D). These results suggest that activation of STAT3 during H5N1/483 infection inhibits apoptosis. It has been previously shown that H5N1 NS1 protein, when over-expressed in HeLa cells reduced IFN-beta induced activation of STAT3 and the formation of STAT1:3- and STAT3:3-DNA complex^31^. Our report here demonstrates that by using a live virus to infect AECs, H5N1/483 induces less apoptosis than H1N1/54 and this reduction is due to, at least partially, H5N1/483-activated STAT3.

## Discussion

Influenza A H5N1 infection is associated with extensive lung injury and acute respiratory distress syndrome. Apoptosis of alveolar epithelial cells is observed in the lungs of H5N1 infected patients^5^. Furthermore, necrosis was also reported in highly pathogenic influenza virus infected lung in humans^32^, in mice^33^, and *in vitro* in differentiated bronchial epithelial cell line^15^,^34^. This provided the rationale for our comparison of apoptosis in primary human alveolar and bronchial epithelial cells infected with HPAI H5N1 and seasonal H1N1 influenza viruses. We also used a low pathogenic H9N2/G1 virus which shared a common derivation of the six internal gene segments with H5N1/483 virus, for comparison. While cell necrosis induced by H1N1 and H5N1 viruses was comparable, H1N1/54 virus induced more apoptosis than the two HPAI H5N1 viruses in both cell types. The avian H9N2/G1 virus induced the least necrosis and apoptosis.

Induction of apoptosis by influenza A virus has been studied previously. It has been reported that caspase 3, 8 and 9 are involved in the influenza virus-induced apoptotic cell death^18^,^35^. Gerlach and colleagues reported a comparison between low and highly pathogenic influenza viruses and found that the pandemic H1N1 viruses (A/KY/180/10 and A/KY/136/09) but not the seasonal H1N1 virus(A/BN/59/07), up-regulated the expression of several genes involved in apoptosis^23^. Another study also reports that the gene expression levels of cell death and apoptosis in the mouse lung were elevated by HPAI H5N1 and H1N1 virus containing the HA and NA of 1918 pandemic virus but not the H1N1 virus^24^. However, they only compared the gene expression levels but not the mechanisms of the apoptosis pathways. The mRNA expression level may not reflect the activation of the target molecule since signaling molecules do not necessarily require up-regulation for signal transduction. While delayed onset of apoptosis induction by H5N1/483 (A/Hong Kong/483/97) virus compared with seasonal H1N1 (A/Hong Kong/54/98) virus in human macrophages has been reported^21^, others have found that the H1N1 strains (pandemic A/HH/01/09 and seasonal H1N1 A/New Caledonia/20/99) delayed and decreased apoptosis in comparison with H5N1 (A/Thailand/1(Kan-1)/04) virus in the same cell type^36^. These results suggest that viruses from the same subtype may have different properties on apoptosis induction in human macrophages and probably in different cell types. We therefore compared the induction of apoptosis by HPAI H5N1 and low pathogenic H1N1 in primary human AECs. Using the same virus subtype and strain as our previous study^22^, we also found that HPAI H5N1 virus caused delayed apoptosis in AECs when compared to seasonal H1N1 virus. We further dissected the apoptosis induction pathways by two representative viruses by measuring the cleavage of caspases.

In previous studies, influenza A virus-induced apoptosis is caspase-dependent^35^,^37^,^38^,^39^,^40^. Caspase 3, the effector caspase, was activated by both H5N1 and H1N1 viruses in AECs. Consistent with the results of TUNEL staining, the activation of caspase 3 was significantly higher in cells infected with H1N1/54 than H5N1/483. Both the HPAI H5N1 and seasonal H1N1 activated caspase 8 and 9, the extrinsic and intrinsic apoptosis pathway respectively. In addition to caspase-dependent apoptosis, there is caspase-independent apoptosis and apoptosis-inducing factor (AIF) has been found to induce DNA fragmentation once it translocates from mitochondria to the nucleus^41^. However, there was no nuclear translocation of AIF in either H5N1 or H1N1 infected AECs (data not shown) and hence the two viruses do not induce caspase-independent apoptosis.

TRAIL is a pro-apoptotic protein which belongs to the TNF family. Various studies have shown that TRAIL is involved in pathogenesis during viral infection. Zhou *et al*. demonstrated that high expression of TRAIL was detected in primary human macrophages with the infection of highly pathogenic H5N1 virus and triggered apoptosis of T cell through cell-cell interaction^22^. The induction of TRAIL by infiltrating macrophages in the mouse lung after infection of H1N1 (PR/8) is associated with severe pneumonia^42^. Blocking the TRAIL signaling in infiltrating macrophages reduced the severity and mortality suggesting that TRAIL contributes to the lung pathogenesis during severe forms of influenza virus pneumonia. Our study is in line with the above report that HPAI H5N1/483 infection significantly up-regulated TRAIL when compared to low pathogenic H1N1/54 virus and this up-regulation led to H5N1-induced apoptosis but not H1N1. A number of evidence shows that TRAIL is involved in AECs apoptosis and lung injury during severe influenza virus induced pneumonia^42^,^43^,^44^. In addition to induction of apoptosis, TRAIL induces endocytosis of Na, K-ATPase by AECs^45^ and lung damage by TRAIL has been reported to facilitate secondary bacterial invasion^46^. Our study demonstrated that TRAIL is also expressed by AECs as well as in macrophages, as reported by Herold’s group^42^. These results indicate that AECs infected with H5N1 contribute to pathogenesis by production of TRAIL in the lung. A recent study reports that type I IFN induced by influenza infection in mice, leads to up-regulation of TRAIL and DR5. Interruption of TRAIL-DR5 interaction protects influenza infection in 129 mice^44^ pointing out the role of DR5 in severe influenza disease, which has not been investigated in this study.

The relationship between apoptosis and replication of influenza virus is still not clear. Previous study demonstrated that supplementing pro-caspase 3 to a cell line without caspase 3 elevated the virus titer by 30 folds^37^. These results suggest that caspase 3 activation is required for replication of influenza virus^37^. A number of studies on the regulatory pathways of influenza-induced apoptosis reveals that a timely control of pro- and anti-apoptotic signals is important for influenza viral replication in host cells^47^,^48^,^49^. However, despite of the delay in apoptosis induction by H5N1 compared to H1N1, the infectivity and replication kinetics of all the influenza viruses were comparable suggesting that the rate of apoptosis does not contribute to the virus replication in AECs.

Another unresolved question is how H5N1 virus delays apoptosis. Different groups attempt to explain this by investigating the viral factors and apoptosis induction. Several influenza viral proteins have been shown to be able to regulate apoptosis of the target cells when they are expressed in isolation. Lam *et al*. reported that NS1 protein of HPAI H5N1 induced more apoptosis in bronchial epithelial cell line than NS1 protein from seasonal H1N1 and H3N2 influenza viruses^50^. However, Chen *et al*. found that H5N1 PB1-F2 unlike H1N1, does not localize to mitochondria and hence has no ability to induce apoptosis^51^. Although their results differentiated the apoptotic induction of the viral proteins from H5N1 and H1N1/H3N2 viruses, contradicting functions among different viral proteins cannot fully address or predict the apoptosis induction by a real virus infection. Using *in vitro* primary cells with live influenza virus infection probably more truly reflects the overall apoptotic responses as a result of interaction between different viral proteins and the host cell. The detailed mechanism by the viral protein of influenza A virus, in particular HPAI H5N1, on triggering the apoptosis remains to be investigated.

Apart from investigating the viral factors, others try to address the question on the host factors. Virus-induced chemokine, CCL5, has been shown to be essential to prevent apoptosis of infected macrophages in mice and in *ex vivo* models after infection with human influenza virus or mouse parainfluenza virus^52^. The protective effect of CCL5 requires the interaction with CCR5 receptor and the activation of PI3K-AKT and MEK-ERK signaling pathways. HPAI H5N1 virus induced strong up-regulation of CCL5 compared to seasonal H1N1 virus in AECs^8^,^28^. These results imply that HPAI H5N1 virus may also delay apoptosis via induction of CCL5 in AECs. Further investigation is needed to confirm the role of CCL5 in apoptosis induction by HPAI H5N1.

Constitutively activated STAT3 has been observed in various types of cancer cell lines and tumors. The activation of STAT3 plays critical roles in cancer formation and STAT3 becomes a target for inducing apoptosis in solid and hematological tumors^25^,^26^,^27^. However, there is only one report on the interaction between H5N1 and STAT3 that over-expression of HPAI H5N1 NS1 protein reduces the IFN-beta induced formation of STAT1:1, STAT1:3 and STAT3:3-DNA complexes and hence disrupts interferon signaling in HeLa cells^31^. In this study, we monitored the activation of STAT3 by both H5N1 and H1N1 virus infection in AECs and found that H5N1 induced more phosphorylated form of STAT3 than H1N1. More importantly, we demonstrate that H5N1 induced more apoptosis when STAT3 was transiently knocked-down in AECs. These results reveal that HPAI H5N1-delayed apoptosis is via the activation of STAT3. The discrepancy may be due to the fact that while Jia’s group reported the function of the single NS1 protein of H5N1, our results demonstrate the outcome of the complicated interactions between different viral proteins and the host proteins during the course of infection.

**Figure 7:**
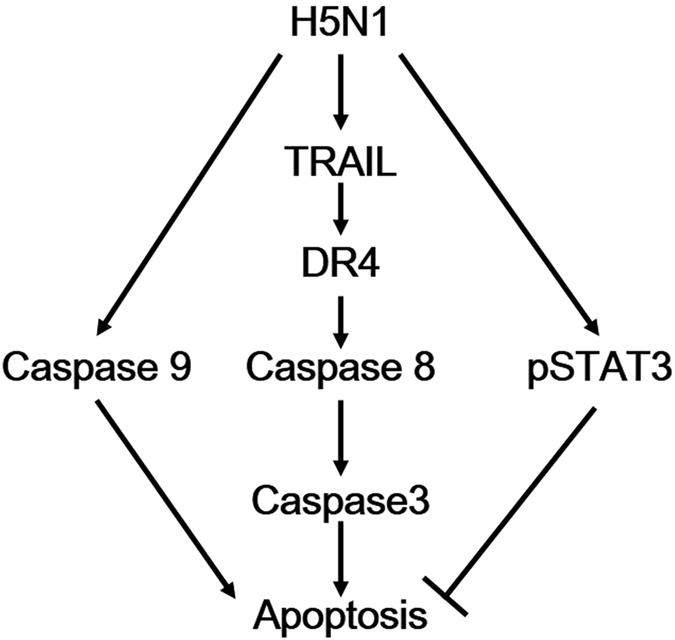
A schematic of the apoptosis induction by HPAI H5N1 virus.

A schematic of apoptosis induction by HPAI H5N1 virus is shown in Fig. 7. We found that H5N1/483 induced apoptosis through activation of caspase 3, 8 and 9. The activation of caspase 8 is probably via the up-regulation of TRAIL and the interaction between TRAIL and DR4, at least partially, leading to the induction of apoptosis. When compared to H1N1, H5N1 activated more STAT3 and subsequently leading to inhibition of apoptosis. The detailed mechanism of how STAT3 regulates the induction of apoptosis in influenza virus needs further investigation. Our identification of STAT3 involved in H5N1-induced apoptosis opens up a new area to explore the role of STAT3 in severe influenza disease and pathogenesis.

Apoptosis has been thought as a host defense mechanism to eliminate the infected cells and help virus clearance. The ability of influenza A virus to delay or prevent the onset of apoptosis potentially contributes to higher pathogenicity. This idea has been supported by a number of studies. A reassortant virus with lower pathogenicity in mice than the wild type virus elicits early and strong induction of apoptosis and interferon^53^. These early events protected the uninfected cells from viral infection. Another study compared resistant (duck) and susceptible (chicken) avian hosts to influenza infection^54^. They found that duck cells underwent more rapid cell death following influenza virus infection than chicken cells. More importantly, a H5N1 virus fatal to ducks did not induce rapid cell death in duck cells. Taken together, these studies and our results here indicate that the ability of H5N1 virus to delay the onset of apoptosis may prolong the time for infected cells to produce infectious progeny viruses, pro-inflammatory cytokines, chemokines^8^,^28^, and TRAIL. These soluble factors in turn can cause more infiltrating immune cells to the lung, which orchestrate to the cytokine storm and the production of TRAIL. At the same time, the generation of pro-inflammatory cytokines and TRAIL causes tissue damage of infected and uninfected cells in the lung. Therefore, the results shown here that the delay of apoptosis by H5N1 compared to seasonal H1N1 virus probably contributes to the pathogenesis of severe influenza disease. In addition, H5N1 delays apoptosis through the activation of STAT3. These results suggest that STAT3 may play heretofore-undescribed critical roles in H5N1 pathogenesis, which needs more detailed investigation.

Our findings provide critical information to further understand the cellular and molecular mechanisms involved in severe human disease associated with H5N1 virus infection, in particular the regulatory pathway of influenza virus induced apoptosis in human respiratory epithelial cells, the major target cells of influenza virus in human. The identification of regulatory pathways and related molecules may contribute to the development of novel drug candidate to alleviate influenza-induced tissue damage.

## Methods

### Ethics statement

Human non-malignant lung tissues were obtained from patients undergoing lung resection in Queen Mary Hospital. Informed consent was obtained from all subjects. The study was carried out in accordance with the guidelines set by The Hong Kong University and Hospital Authority (Hong Kong West) Institutional Review Board (IRB) (Approval no: UW-08355). All experimental protocols were approved by The Hong Kong University and Hospital Authority (Hong Kong West) IRB.

### Influenza virus

Viruses used in these studies included a seasonal H1N1 virus, A/Hong Kong/54/98 (H1N1/54), an avian influenza H9N2 virus, A/Quail/Hong Kong/G1/97 (H9N2/G1) and two HPAI H5N1 viruses isolated from humans, A/Vietnam/1203/04 (H5N1/1203) and A/Hong Kong/483/97 (H5N1/483). These viruses were initially isolated and passaged in Madin-Darby canine kidney (MDCK) cells. Virus titer was determined by using 50% tissue culture infectious dose (TCID_50_) in MDCK cells in the presence of 1 μg ml^−1^ of tosylsulfonyl phenylalanylchloromethyl ketone (TPCK)-treated trypsin (Sigma, USA) as previously described^55^.

### Cell culture

Human type I-like alveolar epithelial cells were isolated from human lung tissue as previously described with modification^55^. Briefly, human non-malignant lung tissues were obtained from patients undergoing lung resection in Queen Mary Hospital. Visible bronchi were removed and lung tissue was minced into small pieces. Minced lung tissues were washed, digested, depleted of CD14 positive macrophages, and cultured in small airway growth medium (SAGM) (Lonza Walkersville Inc. USA). Cryopreserved primary HBECs with retinoic acid were purchased commercially (Lonza Walkersville Inc., USA). HBECs were cultured with Clonetics bronchial epithelial cells growth medium (BEGM) (Lonza, USA) as previously described^56^.

### Influenza virus infection

HBECs and primary human type I-like alveolar epithelial cells (AECs) were infected with various influenza A viruses at a multiplicity of infection (MOI) of 2. After one hour incubation at 37 °C, the virus inoculum was removed, the cells were washed once with PBS, and replenished with 1 ml of the corresponding culture medium.

### Immunofluorescent staining of influenza viral protein

1 × 10^5^ cells were seeded on cover slips and infected with H1N1/54, H5N1/483, H5N1/1203 and H9N2/G1 influenza virus at a MOI of 2. At 24 hpi, infected cells were fixed with 4% paraformaldehyde (PFA). The cells were permeabilized with 0.2% Triton X solution and incubated with antibody-conjugated with FITC against influenza virus nucleoprotein and matrix protein (Oxoid, UK). The cells were mounted with mounting medium (VECTASHIELD, USA) with 4′, 6-diamidino-2-phenylindole (DAPI) (Vector Laboratories, USA). Infectivity was presented as the percentage of cells with FITC signal.

### Detection of apoptotic cells

Apoptotic cells were detected by using TUNEL (Terminal deoxynucleotidyl transferase biotin-dUTP Nick End Labeling) apoptosis detection kit (Millipore, USA) according to manufacturer’s protocol. In brief, 4% PFA fixed cells were permeabilized with 0.2% Triton-X-100 (Sigma-Aldrich, USA) and incubated with TdT end-labeling cocktail for 1 h at room temperature. The reaction was stopped with TB buffer and the cells were blocked with blocking solution. After incubating with avidin-conjugated with FITC for 30 min at room temperature in dark, the cells were mounted on glass slides with DAPI containing mounting medium (Vector Laboratories Inc, USA). The percentages of apoptotic cells were counted in five fields of each sample using a Nikon epi-fluorescence microscope.

### siRNA mediated gene silencing

siRNAs against human DR4, STAT3 and non-targeting control siRNAs (Thermo Fisher Scientific) were transiently transfected to the cells using HiPerfect transfection reagent (Qiagen). 24 h after transfection, the cells were infected with influenza virus at a MOI of 2 and the percentages of apoptotic cell were detected with TUNEL assay as mentioned above.

### Lactate dehydrogenase (LDH) cytotoxicity detection assay

LDH assay (Clontech, USA) was used to determine the total cell death compared to mock infected cells. Cells were infected by influenza A viruses (H1N1 and H5N1) at MOI 2. Supernatants were collected at various time points and stored at −80 °C until used. 100 μl of culture supernatant was incubated with a mixture of dye (Tetrazolium) and catalyst (diaphorase) (45:1) for 10 minutes at room temperature. The absorbance was measured at 495 nm, with a reference wavelength of 592 nm. Data was expressed as fold change over the absorbance of mock at 24 hpi.

### Real time-PCR

Cell lysates were collected and total RNA was extracted with RNase easy mini kit (Qiagen, USA). cDNA was generated from mRNA with oligo(dT)_12-18_ and Superscript III^TM^ reverse transcriptase (Gibco Invitrogen, USA) according to manufacturer’s protocol. The mRNA expression of target genes was quantified by using Applied Biosystem 7500 Real Time PCR System (Applied Biosystem, USA). Primer sequences and thermal cycles for detection of influenza virus matrix gene (M gene) and β-actin gene have been described previously^57^. The expression of other target genes was quantified and normalized with the expression level of β-actin as described previously^8^,^55^,^56^.

### Western blotting

Cellular lysates (30 μg protein) were subjected to immunoblot analysis with antibodies against cleaved caspase 3, caspase 9, caspase 8, STAT3, phosphorylated-STAT3 (Tyr705) (Cell Signaling Technology), and β-actin (Millipore). Bound antibodies were visualized with respective horseradish peroxidase-coupled anti-rabbit or anti-mouse IgG antibodies and ECL plus detection reagents (GE Healthcare Life Sciences, USA). The intensity of the bands was quantified using Image J. All the quantified bands were normalized with the intensity of β-actin in the corresponding sample.

### Statistical analysis

Experiments using HBECs and AECs were performed independently with at least two different donors. Results were presented as mean ± SEM. Mock infected tissues served as negative controls. The differences in percentages of apoptotic cells, fold change of LDH and log_10_ transformed viral titres over time were compared using two-way ANOVA analysis of variance followed by a Bonferroni multiple-comparison test. The gene expression from real-time PCR, protein level from Western blotting between viruses and the apoptosis induction between control and gene silenced cells was compared using Student’s t test. Values of *P* < 0.05 were considered statistically significant.

## Additional Information

**How to cite this article**: Hui, K. P. Y. *et al*. Highly pathogenic avian influenza H5N1 virus delays apoptotic responses via activation of STAT3. *Sci. Rep.* **6**, 28593; doi: 10.1038/srep28593 (2016).

## Supplementary Material

Supplementary Information
