# Supplementary material for: Highly pathogenic avian influenza H5N1 virus delays apoptotic responses via activation of STAT3
Source: Sci Rep. 2016 Jun 27;6:28593. doi: 10.1038/srep28593 (PMC4921847; doi:10.1038/srep28593)
Supplement: Supplementary Information [file srep28593-s1.pdf]

Highly pathogenic avian influenza H5N1 virus delays apoptotic responses via activation of STAT3

Kenrie PY Hui[1], Hung Sing Li[1], Man Chun Cheung[1], Renee WY Chan[1,2], Kit M Yuen[1], Chris KP Mok[1,3], John M Nicholls[4], JS Malik Peiris[1], Michael CW Chan[1*]

[1]Centre of Influenza Research and School of Public Health, LKS Faculty of Medicine, The University of Hong Kong, Hong Kong SAR, China; [2]Department of Paediatrics, Faculty of Medicine, The Chinese University of Hong Kong, Hong Kong SAR, China; [3]The HKU-Pasteur Research Pole, School of Public Health, LKS Faculty of Medicine, The University of Hong Kong, Hong Kong SAR, China; [4]Department of Pathology, LKS Faculty of Medicine, The University of Hong Kong, Queen Mary Hospital, Hong Kong SAR, China.

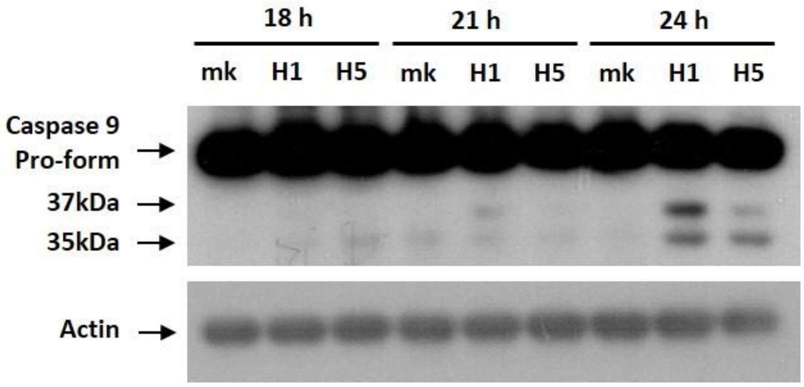

Supplementary Figure S1. Expression of cleaved caspase 9 was detected by Western blotting at 18, 21 and 24 hpi with mock (mk), H1N1/54 (H1) and H5N1/483 (H5) viruses in AECs. The full-length blots of cleaved caspase 9 and actin are shown.

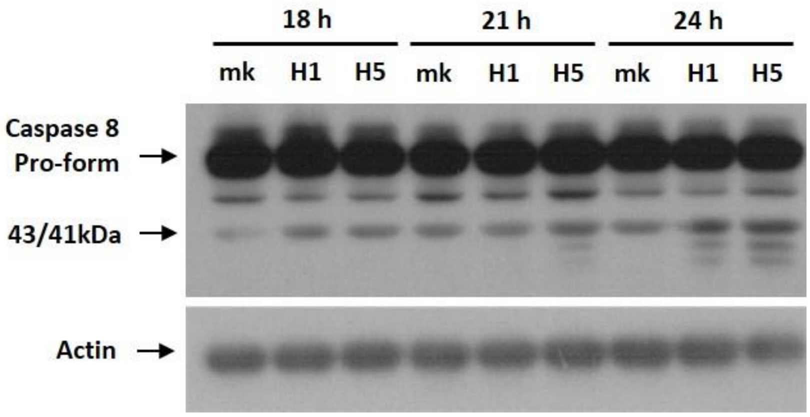

Supplementary Figure S2. Expression of cleaved caspase 8 was detected by Western blotting at 18, 21 and 24 hpi with mock (mk), H1N1/54 (H1) and H5N1/483 (H5) viruses in AECs. The full-length blots of cleaved caspase 8 and actin are shown.
